# Single-Image-Based 3D Reconstruction of Endoscopic Images

**DOI:** 10.3390/jimaging10040082

**Published:** 2024-03-28

**Authors:** Bilal Ahmad, Pål Anders Floor, Ivar Farup, Casper Find Andersen

**Affiliations:** Department of Computer Science, Norwegian University of Science & Technology, 2815 Gjøvik, Norway; paal.anders.floor@ntnu.no (P.A.F.); ivar.farup@ntnu.no (I.F.); casper.andersen@ntnu.no (C.F.A.)

**Keywords:** 3D reconstruction, image enhancement, endoscopy, medical imaging

## Abstract

A wireless capsule endoscope (WCE) is a medical device designed for the examination of the human gastrointestinal (GI) tract. Three-dimensional models based on WCE images can assist in diagnostics by effectively detecting pathology. These 3D models provide gastroenterologists with improved visualization, particularly in areas of specific interest. However, the constraints of WCE, such as lack of controllability, and requiring expensive equipment for operation, which is often unavailable, pose significant challenges when it comes to conducting comprehensive experiments aimed at evaluating the quality of 3D reconstruction from WCE images. In this paper, we employ a single-image-based 3D reconstruction method on an artificial colon captured with an endoscope that behaves like WCE. The shape from shading (SFS) algorithm can reconstruct the 3D shape using a single image. Therefore, it has been employed to reconstruct the 3D shapes of the colon images. The camera of the endoscope has also been subjected to comprehensive geometric and radiometric calibration. Experiments are conducted on well-defined primitive objects to assess the method’s robustness and accuracy. This evaluation involves comparing the reconstructed 3D shapes of primitives with ground truth data, quantified through measurements of root-mean-square error and maximum error. Afterward, the same methodology is applied to recover the geometry of the colon. The results demonstrate that our approach is capable of reconstructing the geometry of the colon captured with a camera with an unknown imaging pipeline and significant noise in the images. The same procedure is applied on WCE images for the purpose of 3D reconstruction. Preliminary results are subsequently generated to illustrate the applicability of our method for reconstructing 3D models from WCE images.

## 1. Introduction

Wireless capsule endoscopy (WCE) was pioneered by Given Imaging in the year 2000 [1]. It offers numerous advantages over traditional endoscopic procedures. It is less invasive, requires no sedation, and offers a painless and comfortable experience for patients. It is used to visually inspect the entire gastrointestinal (GI) tract, from the esophagus to the large intestine, using a small swallowable capsule equipped with a miniature camera. It is used to diagnose inflammatory bowel disease, GI bleeding, and polyps [2]. Despite its many advantages, WCE images also entail several challenges. These include issues related to uneven and low illumination, low resolution, and noise [3]. Moreover, the lack of control over the capsule’s movement within the GI tract restricts the thorough examination of areas of particular interest.

Three-dimensionally (3D)-reconstructed models of WCE images can be effective for conducting a comprehensive analysis of specific areas of interest. By employing 3D reconstruction algorithms, it becomes feasible to transform the 2D images captured by the capsule camera into a 3D representation of the GI tract. Three-dimensional models along with their images can allow gastroenterologists to visualize internal organs from different angles and perspectives, aiding in the identification of abnormalities and facilitating more precise planning for interventions and surgeries. Results in [4] have shown that gastroenterologists find 3D models useful to an extent that they sometimes prefer them over original images.

Within the realm of computer vision, 3D reconstruction poses an intriguing challenge, which requires the utilization of different techniques to image data [5]. Vision-based depth estimation techniques can be classified into different categories. A range of techniques for monocular image-based depth estimation have been developed, including texture gradient analysis [6], image focus analysis [7], and photometric methods [8]. Other approaches leverage multiple images, relying on camera motion or variations in relative camera positions [9]. The integration of 3D reconstruction techniques finds extensive applications across diverse fields, spanning cultural heritage, robotics, medical diagnostics, video surveillance, and more [10,11].

In numerous real-world applications, capturing multiple images of a scene or object from various angles can be challenging. Consequently, single-image-based methods prove effective and suitable in such situations. This is particularly evident in the case of WCE, where the capsule relies on the natural peristaltic contractions to traverse through the human GI tract. Given its low frame rate, it happens that the scene within the GI tract is captured only once. In such circumstances, single-image-based 3D reconstruction techniques are the only viable option.

Shape from shading (SFS) is a method that requires only one image for 3D reconstruction, and therefore, it is a potential candidate for WCE application. Horn and Brooks [12] were among the first to recover the 3D shape of the surface using the SFS method. They obtained surface gradients through an iterative approach relying on a nonlinear first-order partial differential equation (PDE), establishing a relationship between 3D shape and intensity variations within an image. By applying integrability constraints, Frankot and Chellappa [13] demonstrated superior accuracy and efficiency in estimating the depth variable compared with the approach by Horn and Brooks. Kimmel and Sathian [14] employed the numerical scheme based on the fast marching method to recover depth, yielding a numerically consistent, computationally optimal, and practically fast algorithm for the classical SFS problem. Tankus et al. [15] remodeled the SFS method under the framework of perspective projection, expanding its range of potential applications. Similarly, Wu et al. [16] also solved the SFS problem under perspective projection without assuming the light source at the camera center, with a specific focus on medical endoscopy.

The method proposed by Wu et al. [16] closely aligns with the WCE setting, featuring a near-light model with multiple light sources positioned around the camera center. Consequently, we selected their method as a starting point for further experimentation. The methodology follows a two-step process for shape reconstruction. Initially, it involves deriving a reflectance function by considering the relative positions of the light sources, camera, and surface reflectance properties. Following this, the error between the reflectance function and image irradiance is minimized by formulating an image irradiance equation (IIE). While a typical solution to IIE involves an L2 regularizer as a smoothness constraint, we opted for anisotropic diffusion (AD) due to its superior accuracy compared with the L2 regularizer [17].

WCE presents considerable challenges in the domain of 3D reconstruction due to its inherent limitations. The device lacks controllability over its light settings, requiring expensive equipment for operation, which is often unavailable. These practical constraints pose significant challenges when attempting to conduct extensive experiments regarding the assessment of 3D reconstruction quality for WCE images. To address these challenges, we successfully conducted a comprehensive investigation on the 3D reconstruction of synthetic colon images captured with a camera in a virtual environment [4]. In the following experiments, we initially employ images of an artificial colon captured under a controlled environment using an industrial endoscope for the purpose of 3D reconstruction, before transitioning to the analysis of images obtained from WCE. The imaging system of the endoscope behaves like that of WCE, though it introduces significantly less lens distortion. Moreover, it offers higher resolution than a typical WCE image, and the light strength can be manually controlled. The endoscope has six rectangular-shaped light-emitting diodes (LEDs) surrounding the camera behind a protective glass covering. The known dimensions of the artificial colon provide a reference for assessing the correctness of the reconstructed 3D colon model.

**Figure 1:**
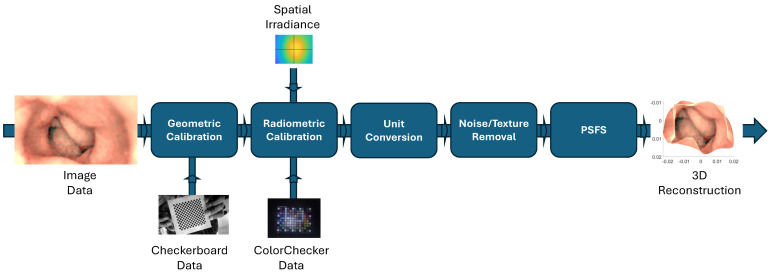
Comprehensive pipeline for 3D reconstruction using PSFS algorithm.

This article utilizes a single-image-based method to reconstruct the 3D shape of the artificial colon. The camera is corrected for lens distortion, and the light source intensity of the endoscope has also been measured. The camera response function (CRF) is estimated to convert the device’s output grayscale image to image irradiance. The method proposed by Andersen et al. [18] is employed, which uses a single image of a ColorChecker to compute the CRF of a camera with an unknown imaging pipeline. Wu et al. [16] assume an ideal multiple-point light model in their PSFS approach. Given that the endoscope is equipped with six light sources, it should closely align with the characteristics of the ideal six-point light model. However, the endoscope light sources produce a different pattern due to their rectangular shape and the presence of a glass covering, which can lead to scattering and interference effects. Therefore, corrections are applied to the captured image to account for this deviation. Thereafter, the near-light perspective SFS (PSFS) algorithm that integrates AD as a smoothness constraint is applied to reconstruct the 3D shapes of the endoscopic images. The PSFS algorithm utilizes grayscale images. Therefore, the albedo is simply a reflection factor between 0 and 1. Initially, well-defined primitive objects are tested to assess the method’s robustness and accuracy. Afterward, the same methodology is applied to recover the geometry of the colon. The known dimensions of the artificial colon also provide a reference for assessing the correctness of the reconstructed 3D colon model. In the end, we present preliminary results of 3D reconstruction using PillCam images, illustrating the potential applicability of our method across various endoscopic devices. The core contributions of the paper are as follows:

- We present a comprehensive pipeline for step-by-step 3D reconstruction using an AD-based PSFS algorithm, as demonstrated in Figure 1. This pipeline is generic and applicable to any endoscopic device, provided that we have access to the required image data for 3D reconstruction, as well as data for geometric and radiometric calibration.
- We utilized JPG images and opted for an endoscope where access to RAW image data was unavailable, reflecting real-world scenarios where RAW data may not be accessible. This choice underscores the practical applicability of our approach, as in many real-world applications, access to RAW data is limited.
- We validated the AD-based PSFS method in real-world scenarios by conducting 3D reconstruction on simple primitives and comparing the results with ground truth—a practice seldom addressed in the literature. This rigorous validation process enhances the credibility and reliability of our approach.
- We present simple methods for estimating the spatial irradiance and light source intensity of the endoscope, designed for scenarios where relying on multiple images for radiometric calibration is not feasible. Further details on these methods are provided in Section 2.4 of the article.

The rest of the article is organized as follows: Section 2 provides an overview of various methodologies for 3D reconstruction, encompassing the PSFS model with anisotropic diffusion, geometric and radiometric calibration of the endoscope, albedo measurement, image rescaling, and denoising. Section 3 details the entire experimental setup, beginning with the creation of ground truth models, followed by image capture, and concluding with the reconstruction of 3D surfaces for primitives and an artificial colon. Additionally, preliminary results for WCE images are presented. Lastly, Section 4 concludes the article.

## 2. Methods Overview

This section covers various methods involved in 3D reconstruction using the PSFS method with an output image from an endoscope. We begin by introducing the PSFS method with AD (Section 2.1). Following that, we discuss the different calibration and preprocessing steps necessary before inputting the image into the PSFS algorithm. Initially, geometric calibration of the endoscope is conducted by capturing images of a checkerboard to correct distortion and determine camera intrinsic parameters, such as focal length (Section 2.2). Subsequently, the captured endoscopic image intended for 3D reconstruction undergoes radiometric calibration, involving the computation of CRF and spatial irradiance (Section 2.4). The radiometrically corrected image is then rescaled (Section 2.5) and denoised (Section 2.6). The comprehensive pipeline of the 3D reconstruction algorithm using the PSFS method is illustrated in Figure 1.

### 2.1. PSFS Model

**Figure 2:**
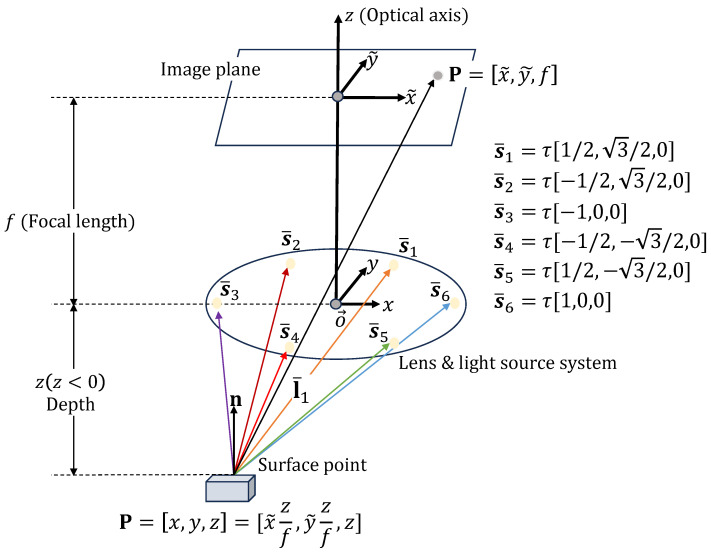
PSFS model with light source at the camera center O. (x,y,z) represents the camera coordinate system, which is centered at O. The *z*-axis is the optical axis, pointing towards the image plane.

In this section, we cover the PSFS method, where six-point light sources are placed around a camera and the camera is directed towards the negative z-axis, as shown in Figure 2. Under perspective projection, the relationship between image coordinates $\left(\tilde{x},\tilde{y}\right)$ and the camera coordinates (x,y,z) is given as follows:

(1) $$x=\tilde{x}\frac{z}{f}\;y=\tilde{y}\frac{z}{f},$$

where *f* denotes the camera’s focal length. Assuming a diffuse surface, the reflected light from the point P can be determined using Lambert’s cosine law and inverse square fall-off law from multiple light sources as follows [16]:

(2) $$R\left(\tilde{x},\tilde{y},z,p,q\right)=I_{o}\rho \sum_{i=1}^{6}\left(\frac{n\left(\tilde{x},\tilde{y},z,p,q\right)\cdot l_{i}\left(\tilde{x},\tilde{y},z\right)}{r_{i}{\left(\tilde{x},\tilde{y},z\right)}^{2}}\right),$$

where $I_{o}$ represents the intensity of the light source(s), ρ denotes the albedo of the surface, and *p* and *q* are the surface gradient components along the *x* and *y* directions, respectively. Furthermore, $r_{i}{\left(\tilde{x},\tilde{y},z\right)}^{2}$ accounts for the inverse square fall-off distance from each point light source, $l_{i}$ is a unit vector aligned along the $i^{th}$ light ray, and n refers to the surface unit normal, which is computed as follows [12]:

(3) $$n=\frac{\left[-\frac{\partial z}{\partial x},-\frac{\partial z}{\partial y},1\right]}{\sqrt{{\left(\frac{\partial z}{\partial x}\right)}^{2}+{\left(\frac{\partial z}{\partial y}\right)}^{2}+1}}.$$

Given the distance from the camera center to a light source, we can explicitly write the light source vector from the point P as follows:

(4) $${\bar{l}}_{i}=\left[\tau \cos\theta_{i}-\tilde{x}\frac{z}{f},\tau \sin\theta_{i}-\tilde{y}\frac{z}{f},-z\right],$$

where τ is the distance from the camera center to a light source, $\theta_{i}=2\pi i/6$ for i∈[1,6]. The unit vector $l_{i}$ can be expressed as $l_{i}={\bar{l}}_{i}/\left\|{\bar{l}}_{i}\right\|$.

According to Horn and Brooks [12], IIE can be written as follows:

(5) $$R\left(\tilde{x},\tilde{y},z,p,q\right)=I\left(\tilde{x},\tilde{y}\right),$$

where $I(\tilde{x},\tilde{y})$ is the image irradiance. Equation (5) is solved to determine the optimal depth value *z* by minimizing the difference between $I(\tilde{x},\tilde{y})$ and $R(\tilde{x},\tilde{y},z,p,q)$. The optimization equation is established for *z*, while the values of *p* and *q* are updated through the gradients of the modified *z* [17]. The error E(z) is minimized as follows:

(6) $$E\left(z\right)=\lambda e_{i}\left(z\right)+\left(1-\lambda \right)e_{s}\left(z\right),$$

where $e_{i}$ and $e_{s}$ represent irradiance error and smoothness constraint, respectively. λ is a weighting factor and controls the scaling between $e_{i}$ and $e_{s}$. $e_{i}\left(z\right)$ can be computed over the image domain $\Omega \subset R^{2}$ as follows:

(7) $$e_{i}\left(z\right)=\int_{\Omega }{\left(I\left(\tilde{x},\tilde{y}\right)-R\left(\tilde{x},\tilde{y},z,p,q\right)\right)}^{2}d\Omega .$$

$e_{s}\left(z\right)$ is typically a L2 regularizer. However, we have employed AD as a smoothness constraint because it not only enhances the accuracy of the depth map by suppressing noise but also demonstrates effectiveness in preserving structural details of the reconstructed scene, outperforming the L2 regularizer [17,19].

AD is introduced as a smoothness constraint by first calculating a 2×2 structure tensor ($S_{i,j}$) based on the gradient of the depth *z* [20]. $S_{i,j}$ is given as [20] as follows:

(8) $$S_{i,j}=\frac{\partial z}{\partial x^{i}}\frac{\partial z}{\partial y^{j}}.$$

Subsequently, we compute the corresponding eigenvalues $\left(\lambda_{+},\lambda_{-}\right)$ and eigenvectors $\left(\theta_{+},\theta_{-}\right)$ following a similar approach to [21]. Utilizing $\left(\lambda_{+},\lambda_{-}\right)$ and $\left(\theta_{+},\theta_{-}\right)$, the diffusion tensor D is then derived as follows:

(9) $$D=\frac{\partial \psi }{\partial \lambda_{+}}\theta_{+}\theta_{+}^{T}+\frac{\partial \psi }{\partial \lambda_{-}}\theta_{-}\theta_{-}^{T}.$$

In terms of $\left(\lambda_{+},\lambda_{-}\right)$, Lagrangian density ψ can be written as follows [22]:

(10) $$e_{s}\left(z\right)=\int_{\Omega }\psi \left(\lambda_{+},\lambda_{-}\right)d\Omega .$$

Equations (7) and (10) are combined in Equation (6) and can be formulated as follows:

(11) $$E\left(z\right)=\int_{\Omega }\left(\lambda {\left(I-R\right)}^{2}+\left(1-\lambda \right)\psi \left(\lambda_{+},\lambda_{-}\right)\right)d\Omega .$$

The solution to Equation (11) is given by Euler–Lagrange PDE:

(12) $$\lambda \left(I-R\right)\frac{\partial R}{\partial z}+\left(1-\lambda \right)\nabla \cdot \left(D\nabla z\right)=0,$$

which we numerically solve by gradient descent:

(13) $$\frac{\partial z}{\partial t}=\nabla \cdot \left(D\nabla z\right)+\frac{\lambda }{1-\lambda }\left(I-R\right)\frac{\partial R}{\partial z}.$$

Similar to [17], $I(\tilde{x},\tilde{y})$ is utilized to derive the structure tensor. Through this single-step computation of the structure tensor, the process becomes efficient, making the computation task simpler and more linear.

### 2.2. Geometric Calibration

Geometric calibration is needed to estimate the camera’s intrinsic parameters as well as its lens distortion. It has been observed that the endoscope exhibits minimal lens distortion towards its periphery. However, the necessity arises to rectify this distortion for the sake of precise depth estimation, as the SFS algorithm assumes a pinhole model.

For geometric calibration, we employed a standard checkerboard measuring 10×10 cm, with each individual square on the board measuring 4 mm. The images are taken at a 10 cm distance from the tip of the camera at different angles. The MATLAB camera calibration toolbox is used for the geometric calibration of the endoscope [23]. The intrinsic parameters are computed using Heikkila’s method [24] with two extra distortion coefficients corresponding to tangential distortion.

**Figure 3:**
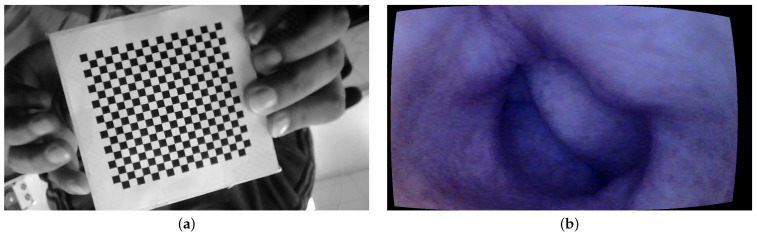
Geometric calibration: (**a**) checkerboard and (**b**) geometric calibration.

The MATLAB camera calibration toolbox basically requires between 10 and 20 images of the checkerboard from different viewing angles. A total of 15 images of the checkerboard are used in our case. An image of the checkerboard is shown in Figure 3a. The camera model is set to standard, and radial distortion is set to 2 coefficients as it is observed that the endoscope camera has little distortions towards the periphery. Figure 3b shows a sample image of the colon corrected for lens distortion.

It is important to mention here that the procedure is repeated three times with three different sets of checkerboard images to confirm the consistency in the results. The estimated focal length is around 2.4±0.1 mm for all three sets, and there is no skew observed.

### 2.3. Albedo Measurement

Albedo is the fraction of incident light that a surface reflects. It has a value between 0 and 1, where 0 corresponds to all the incident light being absorbed by the surface and 1 corresponds to a body that reflects all incident light. The primitives have diffused white surfaces. Therefore, the albedo is assumed to be ρ=1 for all the primitive objects.

**Figure 4:**
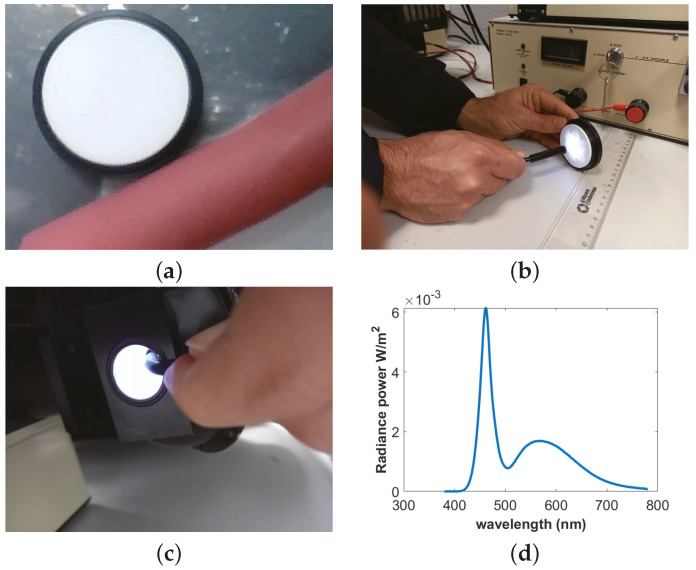
Radiance intensity and albedo measurement: (**a**) albedo, (**b**) nonisotropic light, (**c**) uniform light, and (**d**) radiance power.

The artificial colon consists of a soft rubber material with a nearly uniform pinkish color. Therefore, it is necessary to measure the albedo of the surface. The albedo of the colon is measured by taking the image of the colon and a diffuse spectralon tile placed side by side. Both the spectralon and the colon are kept at an equal distance from the camera, and an image is taken outside so that both surfaces have a uniform distribution of light, as shown in Figure 4a. The albedo of the surface is measured by taking the ratio between the colon and the spectralon pixel value at any given location. The estimated albedo value of the artificial colon is ρ=0.60.

### 2.4. Radiometric Calibration

Radiometric calibration has been performed to measure the light intensity, CRF, and spatial distribution of the light intensity on the image. The PSFS algorithm assumes a pinhole model with ideal multiple-point light sources. Therefore, it is crucial to convert from a grayscale image to image irradiance via CRF and correct for the anisotropy of the light source [16], as discussed in Section 2.4.2. Section 2.4.2 and Section 2.4.3 provide detailed discussions on the CRF estimation and anisotropy correction, respectively. Measuring light source intensity is also important, as it is a crucial parameter for computing the reflection function given in Equation (2).

#### 2.4.1. Light Source Intensity Measurement

The light intensity of the endoscope is measured by using a CS2000 spectroradiometer [25]. An integrating sphere (IS) must be used to measure intensity because of the nonisotropic behavior of the light source. The IS is a hollow spherical cavity with its interior coated with diffused white reflective material. The aim of the integrating sphere is to provide a stable and uniform illumination condition. An endoscope is placed inside the IS, and radiance power *P* is measured over the visible spectrum. After measuring the solid angle ω of the endoscope light, $I_{o}$ is calculated as follows: $I_{o}=P/\left(4\pi \right)\times \omega$. The nonuniformity of the light source, the uniformity of the endoscope light inside the IS, and spectra of the light are shown in Figure 4b–d, respectively.

#### 2.4.2. Camera Response Function

CRF is essential to convert the device’s output grayscale image to image irradiance [16]:

(14) $$I\left(\tilde{x},\tilde{y}\right)=\frac{r^{-1}\left[\upsilon \left(\tilde{x},\tilde{y}\right)\right]}{M(\tilde{x},\tilde{y})},$$

where $I(\tilde{x},\tilde{y})$ is the image irradiance, $\upsilon (\tilde{x},\tilde{y})$ is the grayscale image, and r(·) is the CRF. $M(\tilde{x},\tilde{y})$ incorporates the deviation from the ideal point-light source assumed by PSFS.

The endoscope used in this work has an unknown imaging processing chain, and there are no means of controlling the exposure time. This decision was intentional, reflecting the common limitation among WCE devices available in the market, which generally do not offer any control over the exposure time. By selecting an endoscope that mimics the behavior of typical WCE devices, our approach demonstrates applicability to a broader range of endoscopic devices.

Through experimental observations with the endoscope, it has been observed that the camera performs automatic exposure adjustments. During the image capture process of the SG ColorChecker [26], we have further noted the camera’s automatic color adjustment and white balancing mechanisms in operation. It is worth noting that this is similar to the functionality of a standard WCE. These complicating factors have compelled us to abstain from methods that utilize multiple images for the estimation of the CRF.

**Figure 5:**
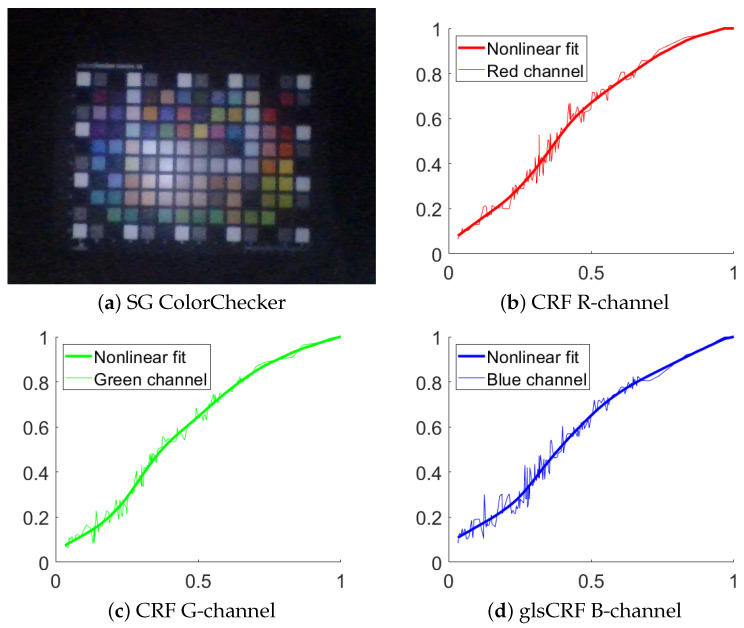
Results of camera response function. (**a**) Image of SG chart captured with an endoscope and used for estimating the CRF and the light distribution. (**b**) CRF in red channel. The red dotted line represents the data point. The red line represents the nonlinear fit. The horizontal axis represents the normalized image intensity, and the vertical axis represents the normalized image irradiance, the same in (**c**,**d**). (**c**) The green dotted red line represents the data point. The green line represents the nonlinear fit. (**d**) The blue dotted line represents the data point. The blue line represents the nonlinear fit.

The method by Andersen et al. [18] is applied to measure the CRF. The method requires only a single image of a ColorChecker to estimate volumetric, spatial, and per-channel nonlinearities. These nonlinearities involve compensating for both physical scene and camera properties through a series of successive signal transformations, bridging the gap between the estimated linear and recorded responses. The estimation process relies on a novel principle of additivity, computed using the spectral reflectances of the colored patches on the ColorChecker. The SG ColorChecker [26] is used to estimate the CRF. An image of the ColorChecker from endoscope and camera response curves is shown in Figure 5.

#### 2.4.3. Spatial Irradiance

The reflection model mentioned in the PSFS method is based on six-point light sources and demands an ideal six-point light distribution in the image to correctly determine the 3D geometry. The endoscope light deviates from an ideal six-point light distribution model due to the rectangular shape of the light sources and the scattering and interference effect caused by the glass on top of the endoscope lens. An inclination in the light sources has been detected, and also, due to the presence of six noncentral light sources, we observe a deviation where the maximum intensity does not align precisely with the image center. Therefore, it is important to quantify these additional effects and compensate for them so that the resulting reflection model satisfies the conditions of six-point light sources. According to [16],

(15) $$\tilde{M}\left(\tilde{x},\tilde{y}\right)=M\left(\tilde{x},\tilde{y}\right)\cdot \sum_{i=1}^{6}\frac{n\cdot l_{i}}{r_{i}^{2}},$$

where the second term on the right side in Equation (15) represents the light distribution from the six-point light sources.

**Figure 6:**
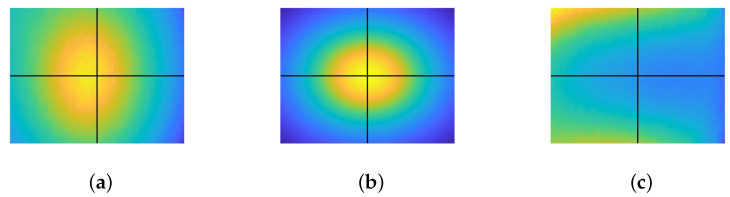
Correction of light distribution. The point where horizontal and vertical lines intersect denotes the image center: (**a**) $\tilde{M}\left(\tilde{x},\tilde{y}\right)$, (**b**) $\sum_{i=1}^{6}\frac{n\cdot l_{i}}{r_{i}^{2}}$, and (**c**) $M\left(\tilde{x},\tilde{y}\right)=\tilde{M}\left(\tilde{x},\tilde{y}\right)/\sum_{i=1}^{6}\frac{n\cdot l_{i}}{r_{i}^{2}}$.

An image of a white diffuse paper, considered as $\tilde{M}\left(\tilde{x},\tilde{y}\right)$ in our context, was captured and is displayed in Figure 6a. It is noticeable from the image that the endoscopic lighting deviates from the ideal six-point light configuration, exhibiting an oval pattern with an offset from the image center. The ideal six-point light distribution model is constructed by physically measuring the distance from the diffused paper to the tip of the endoscope, as shown in Figure 6b. Finally, $M(\tilde{x},\tilde{y})$ is recovered using Equation (15) and then compensated for in the image. $M(\tilde{x},\tilde{y})$ is shown in Figure 6c.

### 2.5. Unit Conversion

The parameters computed thus far are in physical units, leading to the estimation of *R* in physical coordinates. To establish a consistency between $I(\tilde{x},\tilde{y})$ and *R*, as outlined in Equation (13), $I(\tilde{x},\tilde{y})$ is transformed from pixel units to physical units. This conversion is achieved as follows:

(16) $$I_{p}\left(\tilde{x},\tilde{y}\right)=\frac{I\left(\tilde{x},\tilde{y}\right)-\min I\left(\tilde{x},\tilde{y}\right)}{\max I\left(\tilde{x},\tilde{y}\right)-\min I\left(\tilde{x},\tilde{y}\right)}\times \left(I_{o}\rho \frac{\cos\theta }{r^{2}}\right),$$

where $I_{p}\left(\tilde{x},\tilde{y}\right)$ denotes the physical value of the image irradiance. θ is the angle between the surface normal and the light ray at the point on the surface where illumination is maximized. *r* is the distance from the light source to the point on the surface where illumination is maximized. In the case of the primitives, the points are measured, whereas, in the case of the colon, the estimation of the parameters *r* and θ relies on factors such as the field of view (FOV) of the camera, the total length of the colon, and the position of the camera within the colon.

### 2.6. Image Denoising

In endoscope images, significant noise is observed, mainly due to JPEG compression artifacts. These artifacts include blocky patterns and color distortions. A noisy image when fed into the SFS algorithms can destabilize the differential equations due to inaccuracies and ambiguities in shading information, which can lead to inaccuracies in the estimation of surface normals and object shape.

In order to reduce the noise, the method by Xu et al. is utilized [27]. The method essentially separates the visual information related to the surface texture of an object from its underlying structural components within an image. We employ this method to remove noise from the image while retaining its structural details. The method is based on the relative total variation scheme, which captures the essential difference between texture and structure by utilizing their different properties. Later, they employed an optimization method that leverages novel variation measures, including inherent variation and relative total variation, to identify significant structures while disregarding the underlying texture patterns.

### 2.7. Assessment Criteria

The reconstructed 3D shapes of the different primitives are compared with ground truth models by measuring relative root-mean-square error ($r_{\mathrm{RMSE}}$) and relative max-depth error ($r_{\mathrm{MDE}}$). These metrics are chosen to quantify depth errors with respect to a reference depth, making the results easily interpretable. $r_{\mathrm{RMSE}}$ quantifies the overall geometric deformation present in the reconstructed 3D model, while $r_{\mathrm{MDE}}$ highlights the maximum deviation observed between the 3D-reconstructed model and the ground truth.

$r_{\mathrm{RMSE}}$ allows for the evaluation of geometric distortion in the 3D-reconstructed model. A perfect 3D reconstruction is indicated by an error value of 0, whereas a highly distorted 3D reconstruction corresponds to a value of 1. $r_{\mathrm{RMSE}}$ is computed as follows:

(17) $$r_{\mathrm{RMSE}}=\frac{1}{d_{\max}}\sqrt{\frac{1}{n}\sum_{i=1}^{n}|\hat{D_{i}}-D_{i}{|}^{2}},$$

where *D*, $d_{\max}$, $\hat{D}$, and *n* represent ground truth depth, maximum ground truth depth point, depth of the recovered 3D shape, and total number of depth points considered for error estimation, respectively.

$r_{\mathrm{MDE}}$ indicates the relative maximum deviation between the estimated depth values produced by a 3D reconstruction algorithm and the ground truth depth values. A low $r_{\mathrm{MDE}}$ suggests that the majority of depth estimates are close to their ground truth counterparts, indicating high accuracy in the 3D reconstruction. Conversely, a high $r_{\mathrm{MDE}}$ implies significant discrepancies between the estimated and ground truth depth values, indicating poorer accuracy in some places in the reconstruction. $r_{\mathrm{MDE}}$ is computed as follows:

(18) $$r_{\mathrm{MDE}}=\frac{1}{d_{\max}}\max|\hat{D_{i}}-D_{i}|.$$

## 3. Experiments and Results

### 3.1. Ground Truth Models

The primary goal of performing depth estimation on simple primitives is to gauge the accuracy and effectiveness of the PSFS method on endoscopic images. This evaluation was conducted in the context of a camera system that contains an unknown imaging pipeline, and where the captured images exhibit significant noise. This approach, involving the reconstruction of fundamental geometric shapes and their subsequent comparison with ground truth models, will prove effective in achieving the desired evaluation.

The experiments are conducted on geometric primitives with known dimensions, including a sphere, a cube, and a pyramid. These primitives have a diffuse surface with an albedo ρ≈1. Given that these primitives have well-defined geometry and known dimensions, ground truth models of these three primitives are generated in MATLAB to compare them with reconstructed surfaces.

### 3.2. Image Acquisition

**Figure 7:**
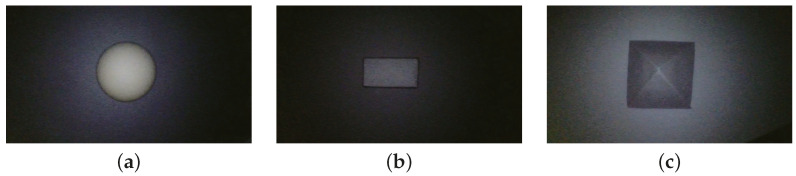
Images of primitives captured with an endoscope: (**a**) sphere, (**b**) cube, and (**c**) pyramid.

The PSFS algorithm is subjected to comprehensive testing using a variety of images, including synthetic colon images and different geometric primitives of known dimensions. The images of the primitives are captured by placing a diffuse paper beneath them to ensure a uniform albedo throughout the scene. A series of images capturing these different primitives are presented in Figure 7.

**Figure 8:**
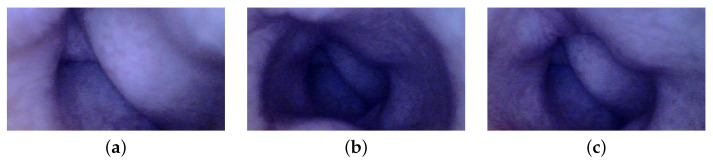
Images of artificial colon captured with an endoscope: (**a**) ROI-1, (**b**) ROI-2, and (**c**) ROI-3.

**Figure 9:**
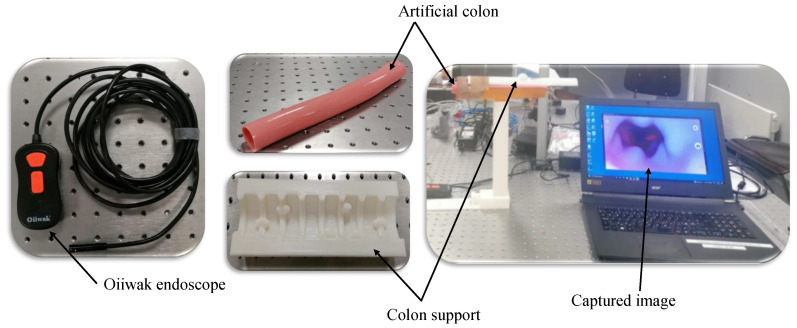
Setup and equipment used for image acquisition of synthetic colon.

Additionally, the images of the synthetic colon are acquired to assess the method’s applicability for potential 3D reconstruction applications within the context of WCE. A synthetic colon [28] is an artificial phantom of a colon without deformation and has a smooth wall with a diameter and length of 0.028 m and 0.3 m, respectively. Therefore, a deformed support [29] is used to hold this colon and produce deformations similar to a real colon. The colon is placed in its support, and one of the ends is closed with a clip. The endoscope is inserted from the other end, and a series of images of the deformed colon are captured, as shown in Figure 8. The endoscope used in the experiment is an *Oiiwak WiFi endoscope* [30], which is an industrial endoscope that wirelessly transmits the acquired images or videos to an android device via a software named MoView. Images of the colon, deformed support, endoscope, and setup for capturing images of the artificial colon are shown in Figure 9.

### 3.3. 3D Reconstruction

In the first step, the image captured with the endoscope is corrected for lens distortions. Subsequently, the image undergoes correction by utilizing the CRF and addressing the anisotropy of the six-point light using Equation (14). The image is then converted to physical units using Equation (16). Thereafter, the image is denoised and then input into the PSFS algorithm. A reflectance map is derived using Equation (2) with a flat surface as initial depth *z*. Subsequently, the *z*’s are updated using Equation (13), where the gradients *p* and *q* are computed as $\frac{\partial z}{\partial \tilde{x}}$ and $\frac{\partial z}{\partial \tilde{y}}$, respectively. Notably, the parameter λ assumes different values in distinct cases and is determined empirically within our experimental setup.

**Figure 10:**
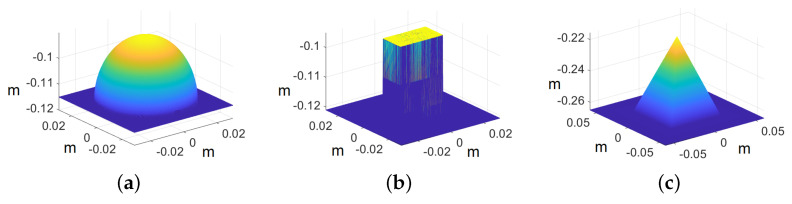
Ground truth models of primitives. The axis represents the values in meters: (**a**) sphere, (**b**) cube, and (**c**) pyramid.

**Figure 11:**
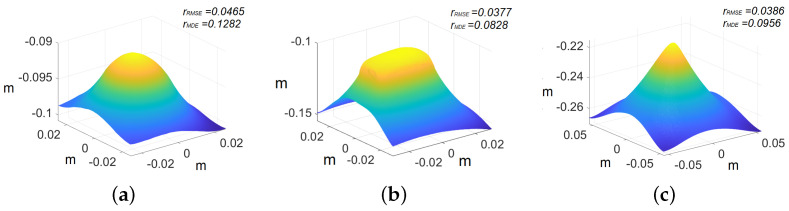
Recovered 3D primitives. The axis represents the values in meters: (**a**) sphere, (**b**) cube, and (**c**) pyramid.

In the case of primitives, the images are cropped to 500×500 pixels because we are interested in recovering the shape of the primitives rather than the whole image. The ground truth models of the primitives are shown in Figure 10, whereas the recovered shape of all the primitives is shown in Figure 11. The reconstructed primitives are compared with ground truth models by computing $r_{\mathrm{RMSE}}$ and $r_{\mathrm{MDE}}$ using Equations (17) and (18), respectively. We achieve $r_{\mathrm{RMSE}}$ at around 0.04 and $r_{\mathrm{MDE}}$ at around 0.10 with respect to ground truth for different primitives, as shown in Table 1.

Table 1 indicates that the sphere exhibits higher errors compared with the pyramid and the cube. This disparity can be attributed to the presence of an inclination in one of the endoscope’s light sources. This manufacturing error poses a significant challenge in accurately modeling the light distribution within our PSFS model. As the sphere covers a larger part of the captured view, in comparison with the other shapes evaluated, the impact of the inclination becomes more pronounced. Consequently, these factors collectively contribute to greater errors in the case of the sphere model.

Full-sized images are used for the reconstruction of the colon model. Color correction is applied to the colon images since their original hue is somewhat pinkish, which appears purplish due to the bluish nature of the endoscope’s lighting. The colon color depicted in Figure 4a serves as the reference color. The difference in hue between the original and the endoscope-captured image of the colon is identified using a chromaticity diagram. Subsequently, the color of the synthetic colon was adjusted to align with its original shade.

**Figure 12:**
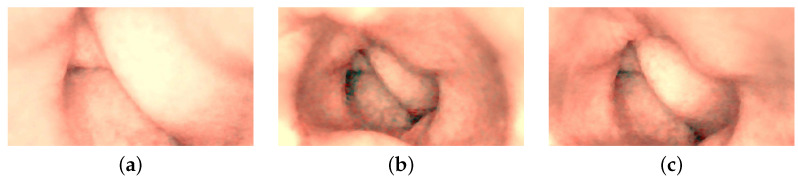
Color-corrected and directionally lit colon images: (**a**) ROI-1, (**b**) ROI-2, and (**c**) ROI-3.

**Figure 13:**
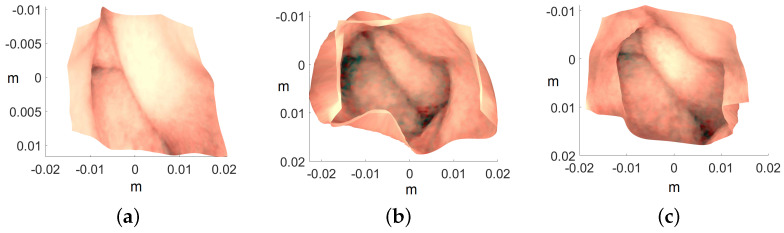
Recovered 3D colon models. The axis represents the values in meters: (**a**) ROI-1, (**b**) ROI-2, and (**c**) ROI-3.

The endoscope is equipped with LEDs, which behave similar to point light sources. This behavior causes dim illumination in deeper regions of the captured images due to the inverse square fall-off law. To address this problem, we have adopted the approach proposed in [4] to enhance contrast, especially in images capturing larger depths. The method involves illuminating the deeper regions by transitioning the lighting in the image from point light to directional light. This transformation is achieved by utilizing surface normals derived from reconstructed 3D models. Following color correction and contrast enhancement on the images of the artificial colon, a notable noise is observed due to the inherent noise in the original images. To address this issue, the enhanced images are further denoised. The enhanced images are converted into their luma and chroma components, with a focus on addressing significant noise present in the luma component. Subsequently, the luma of all the images is subjected to denoising using anisotropic diffusion. The diffusion tensor is derived like in Equation (9), after applying a Gaussian filter to the luma component of the enhanced images, ensuring the preservation of edges in the resulting denoised images. The final geometrically corrected enhanced images of the colon are presented in Figure 12, and the subsequent 3D models, wrapped with enhanced images, are illustrated in Figure 13.

### 3.4. Discussion

The PSFS algorithm demonstrates robustness in handling noisy endoscope-captured images. Initially, the method is tested on simple primitives to assess accuracy by comparing the reconstructions with ground truth models. The results in Table 1 indicate a notable level of accuracy, which, in turn, served as an indicator of the method’s potential for accurately reconstructing the 3D geometry of the colon.

While reconstructing 3D shapes, the number of iterations in the PSFS algorithm varies across experiments. We terminate the process when successive iterations show no significant change in irradiance error $e_{i}\left(z\right)$ according to Equation (7). Throughout the experimentation with the PSFS algorithm, $e_{i}\left(z\right)$ is continuously reduced, indicating, as referenced in [17], an improvement in the quality of depth estimation.

The known dimensions of the artificial colon played a crucial role in assessing the accuracy of the reconstructed 3D colon model during laboratory experimentation. As previously stated, the artificial colon has a diameter of 0.028 m, a value closely matched by all the reconstructed colon models shown in Figure 13. Another significant advantage of employing the endoscope is the extensive laboratory experiments that are challenging to replicate with a WCE in a controlled environment, mainly due to the unavailability of high-cost equipment required for WCE operation. However, after successfully reconstructing the colon shapes using an endoscope that closely mimics the behavior of WCE, confidence is established in the feasibility of applying the same procedure to reconstruct 3D shapes from WCE images.

### 3.5. Preliminary Results of WCE

After experimenting with an endoscope, we subsequently test images of the GI system captured with WCE. These images were acquired during clinical trials involving the examinations of ten patients. The pilot study was conducted in collaboration between Innlandet Hospital Trust and NTNU, Gjøvik, Norway, in 2021, under the consultation of the professor and gastroenterologist Øistein Hovde. The capsule modality used in the examinations was PillCam COLON 2.

**Figure 14:**
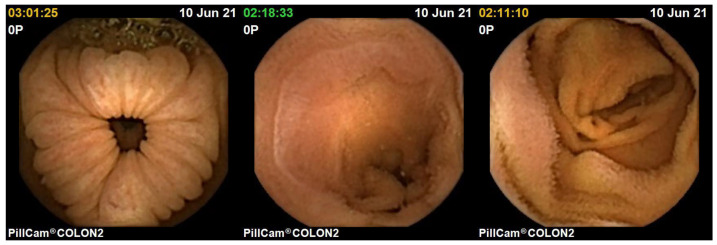
Images of the human colon captured with PillCam COLON 2.

Three distinct images from the colon region of the GI tract are selected, as illustrated in Figure 14. We choose images without any artifacts or deficiencies to demonstrate the applicability of our method on PillCam images. We apply a similar procedure as used with the endoscope, with a notable difference in radiometric calibration due to unavailability of the SG ColorChecker images captured with PillCam. The geometric calibration is performed using images of a checkerboard acquired during the pilot study. For physical unit conversion, *r* and θ are empirically estimated by leveraging the optical properties of WCE, such as effective visibility distance, as provided in the PillCam information manual [31]. Due to the absence of radiometric results, certain assumptions are made for the CRF and the spatial irradiance. It is assumed that the four light sources of PillCam COLON 2 are similar to an ideal four-point light distribution model. The conversion of image intensity values to image irradiance utilizes standard sRGB curves. The mucosal texture of the GI tract is removed using the method proposed by Xu et al. [27] to approximate a uniform albedo throughout the scene. Finally, the PSFS method is employed to reconstruct 3D shapes of the PillCam images.

**Figure 15:**
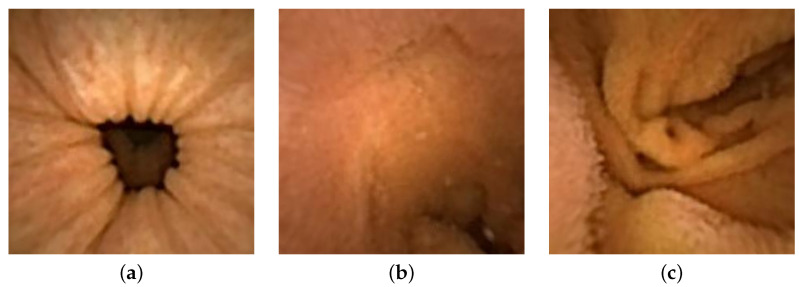
Geometrically corrected cropped images utilized for 3D reconstruction: (**a**) PC-1, (**b**) PC-2, and (**c**) PC-3.

**Figure 16:**
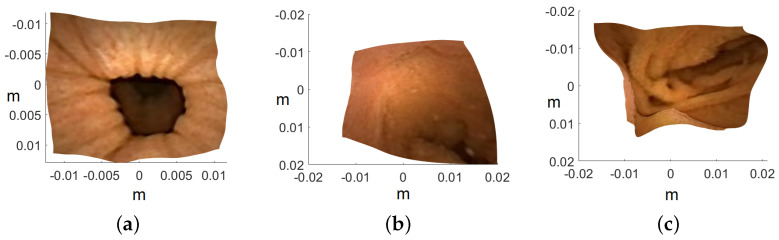
Top view of the recovered 3D human GI regions. The axis represents the values in meters: (**a**) PC-1, (**b**) PC-2, and (**c**) PC-3.

**Figure 17:**
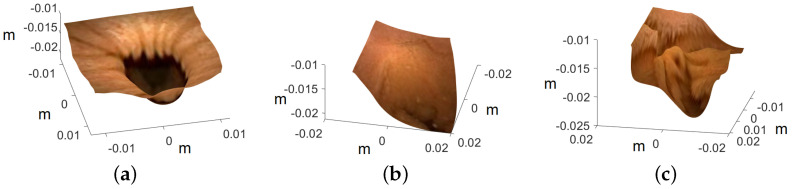
Side view of the recovered 3D human GI regions. The axis represents the values in meters: (**a**) PC-1, (**b**) PC-2, and (**c**) PC-3.

Image are cropped to a size of 275×275, as the MATLAB camera calibration toolbox is unable to handle regions towards the periphery quite well. Cropped images utilized for 3D reconstruction are shown in Figure 15, and the corresponding reconstructed 3D models of all three images are shown in Figure 16. In Figure 17, the side view of all the 3D models are shown, which clearly illustrates that our method successfully reconstructs a significant portion of the structure, even in these preliminary results, despite the absence of radiometric data from the PillCam camera.

The 3D reconstruction results can be further enhanced by conducting radiometric calibration on PillCam, as it is an important parameter to convert the device’s output grayscale image to image irradiance, as per Equation (14). The geometric calibration can be further improved by utilizing other methods that deal with fish-eye lenses [32]. Nevertheless, these preliminary results are quite convincing, demonstrating the capability of our method to handle images with significant lens distortion, even in the absence of radiometric calibration results and albedo values. Further investigation is encouraged to enhance the accuracy of 3D models, as they are recognized as valuable tools during diagnostic assessment in gastroenterology, as highlighted in [4].

## 4. Conclusions

This article investigates the possibility of reconstructing endoscopic images using the PSFS algorithm employed with anisotropic diffusion. Images of simple primitives are initially tested to evaluate the accuracy of the method on endoscopic images by comparing the reconstructed geometries with ground truth models. Afterward, single images of the endoscopes are used to reconstruct the colon surface. Results show that our systematic approach can handle a camera with an unknown imaging pipeline and noisy images and can accurately reconstruct the geometry of the colon.

Additionally, we have implemented a technique utilizing surface normals derived from the 3D-reconstructed models to improve illumination and thereby enhance contrast in images capturing larger depths. This is achieved by changing the illumination in such images from point light to directional light. Various other techniques have also been discussed for the geometric and radiometric calibration of an endoscope camera. This calibration is essential for accurately reconstructing 3D shapes using the PSFS algorithm. In the end, preliminary 3D reconstruction results using PillCam images are provided, demonstrating the potential applicability of our method to different endoscopic devices. In future works, efforts will be made to fully calibrate the PillCam COLON 2 camera to further enhance the 3D reconstruction results.

## Figures and Tables

**Table 1:** Quantitative evaluation for primitives.

Primitives	Cube	Sphere	Pyramid
$r_{\mathrm{RMSE}}$	0.0377	0.0465	0.0386
$r_{\mathrm{MDE}}$	0.0828	0.1282	0.0956

## Data Availability

No new data were created during this study.
